# Probing the Proton-Gated ASIC Channels Using Tetraalkylammonium Ions

**DOI:** 10.3390/biom13111631

**Published:** 2023-11-08

**Authors:** Konstantin K. Evlanenkov, Maxim V. Nikolaev, Natalia N. Potapieva, Konstantin V. Bolshakov, Denis B. Tikhonov

**Affiliations:** Sechenov Institute of Evolutionary Physiology and Biochemistry RAS, St. Petersburg 194223, Russia or konstantin361@mail.ru (K.K.E.); fmedfstud@gmail.com (M.V.N.); or potapieva2004@mail.ru (N.N.P.); or k.bolshakov@biotechnologies.ru (K.V.B.)

**Keywords:** tetraalkylammonium ions, channel block, structure–activity relationship, gating modulation

## Abstract

The action of tetraalkylammonium ions, from tetrametylammonium (TMA) to tetrapentylammonium (TPtA), on the recombinant and native acid-sensing ion channels (ASICs) was studied using the patch-clamp approach. The responses of ASIC1a, ASIC2a, and native heteromeric ASICs were inhibited by TPtA. The peak currents through ASIC3 were unaffected, whereas the steady-state currents were significantly potentiated. This effect was characterized by an EC_50_ value of 1.22 ± 0.12 mM and a maximal effect of 3.2 ± 0.5. The effects of TPtA were voltage-independent but significantly decreased under conditions of strong acidification, which caused saturation of ASIC responses. Molecular modeling predicted TPtA binding in the acidic pocket of closed ASICs. Bound TPtA can prevent acidic pocket collapse through a process involving ASIC activation and desensitization. Tetraethylammonium (TEA) inhibited ASIC1a and native ASICs. The effect was independent of the activating pH but decreased with depolarization, suggesting a pore-blocking mechanism.

## 1. Introduction

Proton-gated ion channels, such as acid-sensing ion channels (ASICs), are widely distributed in the central and peripheral nervous systems. In the brain, they participate in multiple physiological and pathological processes and cause various modulatory effects [1]. Functional ASICs are homo- or heterotrimers formed by ASIC1a/b, ASIC2a/b, and ASIC3 subunits. Only ASIC2b does not form functional homotrimers. All channels are sodium-selective, but ASIC1a-containing channels are also permeable for calcium. ASIC1a, ASIC2a, and ASIC2b are expressed in the brain, whereas ASIC3 and ASIC1b are found in sensory neurons of the peripheral nervous system [1].

Available data on ASIC gating are controversial. Structural studies of chicken ASIC1a suggest that the key step in activation is collapsing of the so-called “acidic pocket”, which is bordered by glutamate and aspartate residues. At high pH values, the acidic residues at the opposite sides of the pocket are deprotonated. Their electrostatic repulsion keeps the pocket in the wide conformation, which corresponds to the closed state of ASIC1a. Lowering the pH causes protonation of acidic residues, and their interaction through shared protons results in the collapse of the pocket and finally channel opening and subsequent desensitization [2].

However, this plausible view apparently conflicts with mutational data, which demonstrate that aspartate and glutamate residues at the acidic pocket are not critical for ASIC gating [3]. Neutralization of the acidic residues in this region does not abolish pH-dependent activation. Moreover, mutational analysis of the low-palm domain revealed another region involved in gating [3]. There are some ASIC homologs, which lack acidic residue in the pocket, but still respond to acidification [4].

The pharmacology of ASICs is a rapidly developing area. Inorganic ions, low-weight organic compounds, and peptides affect ASICs via different mechanisms and modes of action and demonstrate different specificity for ASICs according to the ASIC subunit compositions [5,6,7]. Endogenous ions and organic compounds, lactate [8], histamine [9], and spermine [10] modulate ASICs and affect the ASIC contribution to synaptic transmission [11]. The main mechanisms of ASIC ligand action are pore blocking, modulation of activation, and desensitization. The structure–function relationships of ASIC ligands are complex; consequently, the predictable design of new ligands remains essentially impossible, with rare exceptions. 

One class of compounds known to affect many channel types, including potassium channels and NMDA-type ionotropic glutamate receptors, are tetraalkylammonium ions. Tetraalkylammonium compounds are also known to affect the TRPP3 channel [12], gap junction [13], and even chloride channels [14]. They are among the simplest organic compounds; however, they have a very large spectrum of action and target many proteins. Their simple chemical structure allows rather easy interpretation of structure–function relationships, as demonstrated by classical systematic studies of potassium channels [15,16,17] of the determinants of the “foot-in-the-door” pore block of NMDA receptor channels [18,19]. Thus, tetraalkylammonium ions can serve as important pharmacological probes. For this reason, in the present work, we tested the action of five tetraalkylammonium compounds, tetramethylammonium (TMA), tetraethylammonium (TEA), tetrapropylammonium (TPrA), tetrabutylammonium (TBA), and tetrapenthylammonium (TPtA), on the functioning of native and recombinant ASICs.

## 2. Materials and Methods

The experiments were conducted in accordance with the Rules of the Committee for the Care and Use of Animals of the I.M. Sechenov Institute of Evolutionary Physiology and Biochemistry of the Russian Academy of Sciences, which is fully compatible with the directives of the Council of the European Community 86/609/EEC. Outbred male Wistar rats (12–17 days old, 25–35 g body weight) were obtained from I.M. Sechenov Institute animal facility. After anesthesia with urethane, the rats were decapitated. The brain was quickly taken out and cooled to 2–4 °C in an ice bath. Then, a 7000 smz vibratome (Campden Instruments Ltd., Loughborough, UK) was used to prepare transverse hippocampal slices (250 µm thick), which were stored in a solution containing (in mM) NaCl 124, KCl 5, CaCl_2_ 1.3, MgCl_2_ 2.0, NaHCO_3_ 26, NaH_2_PO_4_ 1.24, and D-glucose 10, aerated with carbogen (95% O2, 5% CO_2_). A vibrodissociation method was used to isolate neurons from the brain slices [20]. Isolated neurons were kept in the solution containing (in mM) NaCl 143, KCl 5, MgCl_2_ 1, CaCl_2_ 2.5, D-glucose 18, HEPES 10, and MES 10 (pH was adjusted to 7.4 with NaOH). The pipette solution contained (in mM) CsF 100, CsCl 40, NaCl 5, CaCl_2_ 0.5, EGTA 5, and HEPES 10 (pH was adjusted to 7.35 with CsOH). We used interneurons from the lacunosum moleculare and radiatum layers of CA1 region, which were shown to express ASIC1a/2a heteromers [21]. These neurons were identified by their specific electrical and morphological characteristics [22]. 

CHO cells were cultured at 37 °C in a humidified atmosphere of 5% CO_2_. Cells were maintained with standard culture conditions (Dulbecco’s modified Eagle’s medium (DMEM) + 10% fetal bovine serum + gentamicin (50 μg/mL). Plasmids encoding green fluorescent protein (GFP) and ASIC subunits were transfected using Lipofectamine 2000 (Invitrogen, Carlsbad, CA, USA) following the manufacturer’s transfection protocol. 

The whole-cell acid-evoked currents were recorded by a patch-clamp technique at a holding potential of −80 mV (unless otherwise stated) using an EPC-10 amplifier (HEKA Electronics, Lambrecht, Germany). Isolated neurons and CHO cells were kept in the solution containing (in mM) NaCl 143, KCl 5, MgCl_2_ 2, CaCl_2_ 2.5, D-glucose 18, HEPES 10, and MES 10 (pH was adjusted to 7.4 with NaOH). The pipette solution contained (in mM) CsF 100, CsCl 40, NaCl 5, CaCl_2_ 0.5, EGTA 5, and HEPES 10 (pH was adjusted to 7.35 with CsOH). Acidic solutions were prepared from extracellular solution by adjusting the pH with HCl immediately before the experiments.

All values are presented as the mean ± standard deviation (SD) from at least five experiments (cells). The significance of the effects was tested with a paired *t*-test (drug vs. control in the same cell). For the data obtained from different cells, the unpaired Student’s *t*-test was used. The effects were considered statistically significant at *p* < 0.05. Molecular docking was performed using Monte Carlo method with energy minimization using ZMM program package (www.zmmsoft.ca).

## 3. Results

**Figure 1:**
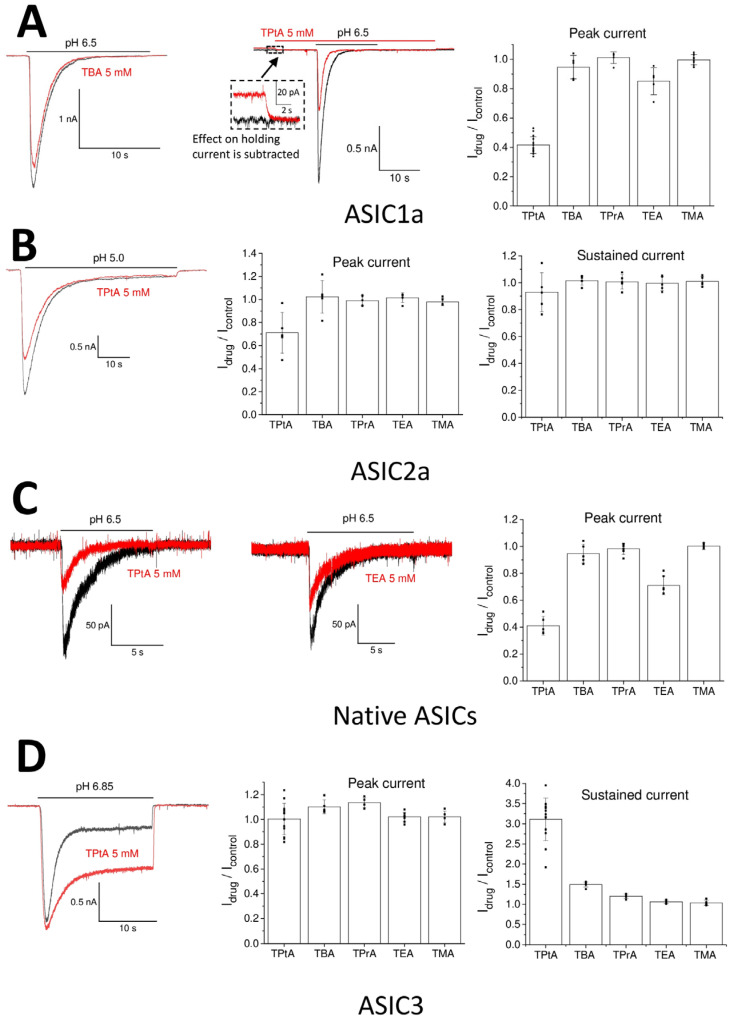
Action of tetraalkylammonium ions on native and recombinant ASICs. Recordings in control and in the presence of drugs are shown black and red, correspondingly. (**A**) Large tetraalkylammonium ions inhibit ASIC1a. Left and middle panels indicate representative recordings of ASIC1a responses in the control (black) and in the presence of 5 mM TBA (left) and TPtA (middle). Middle panel shows extended view of the application protocol. TPtA is applied before the activation and causes non-specific inward current. The responses are aligned to avoid influence of this current on the drug effect (see insert). Right panel presents statistical data. (**B**) Only TPtA inhibits ASIC2a. Left panel shows representative recordings. Middle and right panels present statistical data on peak and sustained components of response. (**C**) Action of tetraalkylammonium ions on native ASICs in rat brain neurons. Left and middle panels, representative recordings of action of 5 mM TPtA and TEA. (**D**) Action of tetraalkylammonium ions on ASIC3. Left panel indicates representative recordings that show that 5 mM TPtA does not affect the peak component and significantly enhances the sustained component of the response. Middle and right panels present statistical data on peak and sustained components of response.

The effects of tetraalkylammonium ions were estimated by activating the ASICs via acidification, which produced 30–50% of the maximum response (pH 6.5 for native ASICs and ASIC1a, pH 5.0 for ASIC2a, and pH 6.85 for ASIC3). At this level of response, the effect on the activation is the most pronounced. The duration of the application was 15 s for ASIC1a and native ASICs, 20 s for ASIC3, and 50 s for ASIC2a due to their different desensitization kinetics. The interval between applications was 40 s to ensure recovery after desensitization in all cases. The compounds were applied at a concentration of 5 mM. Small compounds (TMA, TEA, TPrA, and TBA) did not affect holding currents at −80 mV. By contrast, TPtA caused reversible inward currents of 20 ± 6 pA (n = 11) in neurons and non-transfected CHO cells at neutral pH. The nature of these currents is unknown since tetraalkylammonium ions can affect various conductances. To account for this non-specific effect, after recording the control response to the pH drop, the compounds were applied 10 s prior to the activation and washed out 10 s after the neutral restoring solution. To calculate the effect, the holding current in the presence of a compound was used as a baseline level (Figure 1A, middle panel). 

The action of tetraalkylammonium ions applied at 5 mM concentrations on recombinant and native ASICs is presented in Figure 1. Currents through ASIC1a were insensitive to TMA, TPrA, and TBA (Figure 1A). TEA caused a small but significant decrease in the response amplitude (I_drug_/I_control_ = 0.85 ± 0.09, n = 5, *p* < 0.05). The strongest effect was observed for TPtA (I_drug_/I_control_ = 0.41 ± 0.06, n = 17, *p* < 0.05). The effects of TEA and TPtA were fully reversible. The shape of the response curves for ASIC1a (rise and decay kinetics) was unaffected. ASIC2a was less sensitive to tetraalkylammonium ions (Figure 1B), as only TPtA produced a significant inhibition of the peak current (I_drug_/I_control_ = 0.71 ± 0.18, n = 5, *p* < 0.05). A hint of inhibition of the steady-state component of ASIC2a response was also evident, but the effect was not statistically significant (I_drug_/I_control_ = 0.93 ± 0.14, n = 5, *p* > 0.05). Native ASICs (Figure 1C), which are likely ASIC1/ASIC2 heteromers, qualitatively demonstrated the same sensitivity to tetraalkylammonium ions as was observed for ASIC1a. TEA had a weak inhibitory effect (I_drug_/I_control_ = 0.71 ± 0.07, n = 6, *p* < 0.05), and TPtA inhibited the response more strongly (0.41 ± 0.07, n = 5, *p* < 0.05). The peak component of the ASIC3 responses was insensitive to all tetraalkylammonium ions (Figure 1D). Surprisingly, the steady-state component was markedly potentiated by the bulk tetraalkylammonium ions TBA and TPtA. Only TMA did not have any effect. The effects of TEA and TPrA were small but significant (TEA: I_drug_/I_control_ = 1.06 ± 0.04, n = 5; TPrA: I_drug_/I_control_ = 1.20 ± 0.05, n = 8; *p* < 0.05 for both compounds). All effects were fully reversible.

**Figure 2:**
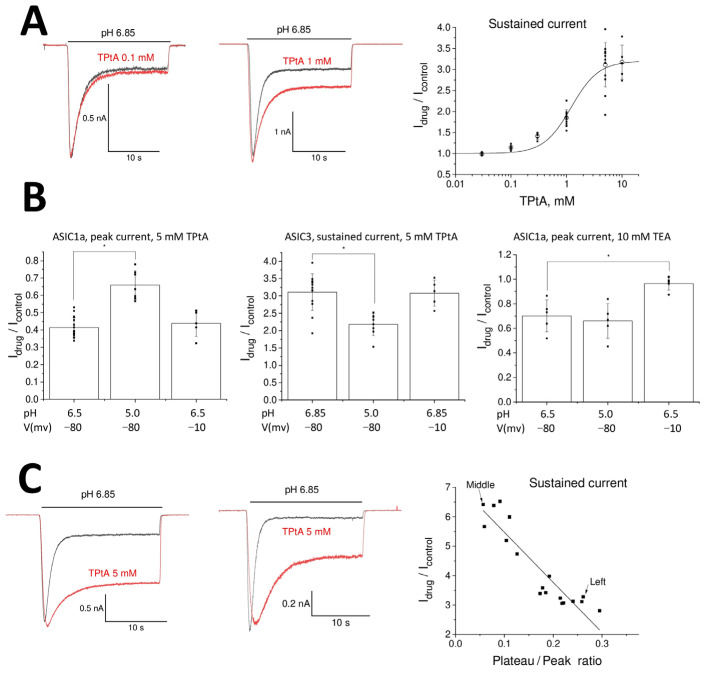
Mechanisms of action of tetraalkylammonium ions on ASICs. Recordings in control and in the presence of drugs are shown black and red, correspondingly. (**A**) Concentration-dependence of TPtA action on the sustained response of ASIC3. Left and middle panels show representative responses. Right panel shows individual data (black squares) and averaged values (open circles), together with the fitting using Hill equation. (**B**) pH- and voltage-dependencies of action. Left, the inhibition of ASIC1a by TPtAa depends on the value of the activating pH but not on the membrane voltage. Middle, the potentiation of ASIC3 sustained current by TPtAa depends on the value of the activating pH but not on the membrane voltage. Right, the inhibition of ASIC1a by TEA does not depend on the value of the activating pH but disappears with membrane depolarization. (**C**) Correlation between the plateau/peak ratio and effect of 5 mM TPtA on the sustained ASIC3 response. Left and middle panels show representative responses. Right panel demonstrates strong dependence between the values.

To further validate the modest effects of TEA and TPrA on ASIC3 receptors, we used them at a 1 mM concentration. At this concentration, the potentiating effects of both compounds disappeared, whereas the effect of TBA remained significant at a 1 mM concentration (I_drug_/I_control_ = 1.12 ± 0.08, n = 6, *p* < 0.05). For TPtA, the most active compound, we analyzed the concentration dependence of its action in greater detail. The experimental design and the limitations of the drug-application system did not allow us to evaluate the whole concentration dependence in a single experiment. Therefore, we pooled the experimental data and fitted it using a Hill equation (Figure 2A). The obtained parameters were as follows: maximal potentiation effect, Emax = 3.2 ± 0.5; half-maximal effective concentration, EC_50_ = 1.22 ± 0.12 mM; and Hill coefficient = 1.8 ± 0.3. 

The opposite actions of tetraalkylammonium ions can arise due to their complex modes of action, which can include, for example, pore blocking, potentiation of activation, and inhibition of desensitization. To discriminate different types of action, we studied the voltage- and pH-dependence of TPtA action on ASIC1a and ASIC3 (Figure 2B, left and middle). In our previous experiments, ASIC1a and ASIC3 were activated by pH drops to 6.5 and 6.85, respectively. These weak acidifications evoke less than 50% of the maximal response. Therefore, we performed an additional experiment with acidification to pH 5.0, which causes almost maximal responses of ASIC1a and ASIC3. The inhibition of ASIC1a responses by 5 mM TPtA decreased from I_drug_/I_control_ = 0.41 ± 0.06, n = 17, at pH 6.5 to I_drug_/I_control_ = 0.66 ± 0.08, n = 7, at pH 5.0. The difference was significant at *p* = 0.05. Potentiation of the ASIC3-mediated steady-state current also significantly decreased from I_drug_/I_control_ = 3.1 ± 0.5, n = 13, at pH 6.85 to I_drug_/I_control_ = 2.2 ± 0.3, n = 8 at pH 5.0. Thus, both ASIC1a inhibition and ASIC3 potentiation by TPtA have similar pH dependence, with the effects becoming weaker at saturating acidifications. By contrast, depolarization from −80 to −10 mV did not cause significant changes in the action of 5 mM TPtA on ASIC1a activated by pH 6.5 (I_drug_/I_control_ = 0.43 ± 0.08, n = 5, *p* > 0.05) or ASIC3 activated by pH 6.85 (I_drug_/I_control_ = 3.08 ± 0.38, n = 5, *p* > 0.05). This lack of voltage dependence is strong evidence against a pore-blocking mechanism of action for these charged compounds. Potentiation of the steady-state response of ASIC3 instead supports an effect on channel desensitization. To clarify the mode of action, we analyzed the relationship between the effect of 5 mM TPtA and the level of desensitization, as measured via the plateau/peak ratio of the control response. Figure 2C shows an unambiguous correlation between a higher plateau/peak ratio in the control and a stronger effect of TPtA on the plateau current. 

The effect of an allosteric modulator is controlled by affinity and efficacy. This is why it is important to reveal which parameter is responsible for the lack of activity of small tetraalkylammonium ions. We studied the action of 5 mM TPtA on ASIC1a and ASIC3 in the presence of the inactive TMA at 5 mM. If TMA does not bind (i.e., it has a weak affinity), it should not affect TPtA action. Contrarily, if the absence of action of TMA is due to weak efficacy, it would compete with TPtA, thereby reducing TPtA action. In both cases (ASIC1a and ASIC3), the effect of TPtA was not changed by the presence of 5 mM TMA (n = 5, *p* < 0.05), suggesting that TMA and presumably other small-size tetraalkylammonium ions have weak affinity. 

The weak but significant activity of TEA on ASIC1a and native ASICs also deserves attention. As with ASIC3 potentiation by TPtA, we analyzed how the inhibitory effect depends on the activating pH and membrane voltage (Figure 2B, right panel). For these experiments, we used a 10 mM concentration to increase the effect. In contrast to the effects seen with TPtA, the inhibition of ASIC1a by TEA was pH-independent (pH 6.5: I_drug_/I_control_ = 0.70 ± 0.13, n = 5; pH 5.0: I_drug_/I_control_ = 0.66 ± 0.14, n = 5). Decreasing the holding voltage from −80 to −10 mV completely abolished the inhibition (I_drug_/I_control_ = 0.97 ± 0.06, n = 5). Thus, the effect of TEA is voltage-dependent, in agreement with the pore-blocking mode of action. 

**Figure 3:**
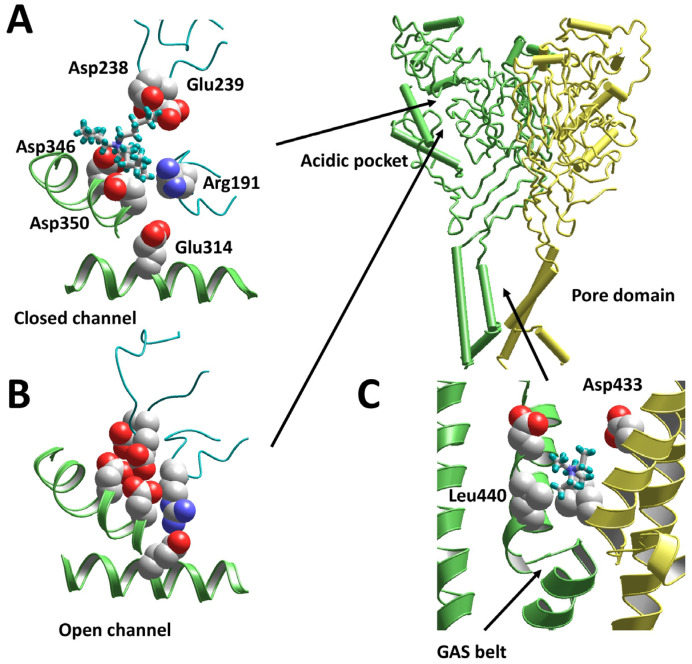
Docking of tetraalkylammonium ions to the ASIC1a models. (**A**) TPtA fits the acidic pocket in the closed state (6vtl). (**B**) In the open state (4ntw), the acidic pocket is collapsed, leaving no room for TPtA binding. (**C**) TEA binds to the open pore (4ntw) at the level of Asp433 just above the GAS belt, which serves as a selectivity filter. Location of the acidic pocket and the pore domain are shown in the whole ASIC1a molecule.

The strong dependence of the activity of tetraalkylammonium ions on their bulkiness suggests that TPtA binds to a rather large pocket in the ASIC protein. Analysis of the available atomic-scale structures allowed us to propose binding to the acidic pocket that can accommodate molecules like TPtA. To test this idea, we built models of closed and open ASIC1a using the structures 6vtl [23] and 4ntw [24], correspondingly. Monte Carlo docking of TPtA demonstrated that it can readily bind in the pocket of the closed channel (Figure 3A). The charge, which is distributed on the surface, provides effective electrostatic interactions with acidic residues at both sides of the pocket. By contrast, in the collapsed conformation of the acidic pocket, which is a feature of open and inactivated channels, the charged residues converge and interact via shared protons, leaving no room to accommodate a TPtA molecule (Figure 3B). Thus, the binding of TPtA can prevent the transition of the acidic pocket from the extended conformation, which corresponds to the closed state, to the collapsed conformation, which corresponds to the open and inactivated states.

The pore-blocking action of TEA was visualized via docking to the open-state model based on the 4ntw structure [24]. Optimal binding occurred above the “GAS belt” at the level of the Asp433 residue, which provides electrostatic interactions with TEA (Figure 3C). Other residues contributing to the interaction energy are Leu440 and Gly432.

## 4. Discussion

Our study used tetraalkylammonium ions as molecular probes of ASICs and revealed important determinants of subtype-specific activation and desensitization gating. We found that large tetraalkylammonium ions inhibit ASIC1a and, to a lesser extent, ASIC2a activation, and attenuate ASIC3 desensitization. Even the most active compound, TPtA, produces strong effects at millimolar concentrations. Therefore, the pharmacological significance of our findings is limited. Tetraalkylammonium ions usually affect other channels in similar concentrations. The exception is the TRPP3 channel, for which TPtA demonstrates an IC_50_ value of about 1 µM [12]. 

Typically, tetraalkylammonium ions cause a steric block of the ion-conducting pores of the channels. We revealed the same mode of action for TEA, which causes voltage-dependent inhibition of ASIC1a. In contrast, the action of TPtA was voltage-independent but decreased under conditions of strong acidifications. Such a mode of action suggests that TPtA affects ASIC1a activation. The enhancing effect on sustained current through ASIC3 also suggests interaction with the gating machinery. 

Docking of the TPtA molecule in the closed-state structure of ASIC1a suggests that it can bind in the acidic pocket. In view of our experimental and modeling results, large tetraalkylammonium ions bind in the acidic pocket in the closed state and sterically prevent its collapse. Our finding that inactive TEA does not compete with TPtA (the action of TPtA did not decrease in the presence of TEA) suggests that small-size compounds are unable to bind to this site. Providing a structural explanation for the enhancing effect of tetraalkylammonium ions on the sustained current through ASIC3 is more difficult. This channel subtype is much less studied than ASIC1a; in particular, the atomic-scale structures of ASIC3 are still absent. In view of the controversial data on the acidic pocket’s role in gating (see Introduction), our results indicate that the acidic pocket collapse is not essential for ASIC3 activation but is required for desensitization. Certainly, structural determinants of activation and desensitization in the ASIC family require further investigation, particularly regarding the use of pharmacological agents as molecular probes. 

## Data Availability

Original data are available on request.
